# Effectiveness of Digital Forced-Choice Nudges for Voluntary Data Donation by Health Self-trackers in Germany: Web-Based Experiment

**DOI:** 10.2196/31363

**Published:** 2022-02-21

**Authors:** Katharina Pilgrim, Sabine Bohnet-Joschko

**Affiliations:** 1 Department of Management and Entrepreneurship Faculty of Management, Economics and Society Witten Herdecke University Witten Germany

**Keywords:** quantified self, health self-tracking, digital nudge, data donation, health data, mobile phone

## Abstract

**Background:**

Health self-tracking is an evidence-based approach to optimize health and well-being for personal self-improvement through lifestyle changes. At the same time, user-generated health-related data can be of particular value for (health care) research. As longitudinal data, these data can provide evidence for developing better and new medications, diagnosing rare diseases faster, or treating chronic diseases.

**Objective:**

This quantitative study aims to investigate the impact of digital forced-choice nudges on the willingness of German health self-trackers to donate self-tracked health-related data for research. This study contributes to the body of knowledge on the effectiveness of nonmonetary incentives. Our study enables a gender-specific statement on influencing factors on the voluntary donation of personal health data and, at the same time, on the effectiveness of digital forced-choice nudges within tracking apps.

**Methods:**

We implemented a digital experiment using a web-based questionnaire by graphical manipulation of the Runtastic tracking app interface. We asked 5 groups independently to indicate their willingness to donate tracked data for research. We used a digital forced-choice nudge via a pop-up window, which framed the data donation request with 4 different counter values. We generated the counter values according to the specific target group needs identified from the research literature.

**Results:**

A sample of 919 was generated, of which, 625 (68%) were women and 294 (32%) were men. By dividing the sample into male and female participants, we take into account research on gender differences in privacy tendencies on the web and offline, showing that female participants display higher privacy concerns than male participants. A statistical group comparison shows that with a small effect size (*r*=0.21), men are significantly more likely (*P*=.04) to donate their self-tracked data for research if the need to take on social responsibility is addressed (the prosocial counter value in this case—contributing to society) compared with the control group without counter value. Selfish or pseudoprosocial counter values had no significant effect on willingness to donate health data among male or female health self-trackers in Germany when presented as a forced-choice nudge within a tracking app.

**Conclusions:**

Although surveys regularly reveal an 80% to 95% willingness to donate data on average in the population, our results show that only 41% (377/919) of the health self-trackers would donate their self-collected health data to research. Although selfish motives do not significantly influence willingness to donate, linking data donation to added societal value could significantly increase the likelihood of donating among male self-trackers by 15.5%. Thus, addressing the need to contribute to society promotes the willingness to donate data among male health self-trackers. The implementation of forced-choice framing nudges within tracking apps presented in a pop-up window can add to the accessibility of user-generated health-related data for research.

## Introduction

### Self-tracked Health Data for Research

As of April 2020, more than half a million enthusiastic health self-trackers donated collected data to the federal government research institute responsible for disease control and prevention in Germany, via fitness wristbands or smartwatches [1]. As of February 2021, data records donated have exceeded 170 million sets, including pulse, blood pressure, weight, temperature, physical activity, and information on sleep cycles. Regarding the COVID-19 pandemic, heart rate measurement and physical activity are of particular interest because an accelerated pulse while decreasing activity is a likely fever indicator—a typical COVID-19 symptom. At best, this fever monitor will serve as an early warning system and predict the spread of coronavirus in Germany even before case numbers from public health authorities are available [2].

The approach described is only one instance of how user-generated health-related data could add value to research and support public health measures [3]. Moreover, this kind of real-world data can support the development of pharmaceutical innovation, accelerate rare disease diagnosis, and improve chronic disease treatment [4-10]. For the self-tracked data to be available for research, users have to share, more precisely, donate their data actively to research institutions [11,12]. In addition to observing regulatory, ethical, and privacy issues, it is crucial to know, understand, and evaluate the motives of a specific target group for data donation [13,14]. Research on data donation typically uses a population cross-section sample. Surveys indicate that 4 of 5 Germans are willing to donate their digital health data anonymously and free of charge for medical research [15]. As many as 95% of German and US social media users would specifically donate their data to scientists at universities and public research institutions [15,16]. The results can potentially be highly biased, as individuals may hypothetically signal their willingness to donate data and perhaps not even engage in health self-tracking at all. Thus, on the one hand, research lacks information on the willingness to donate self-tracked health-related data among those who actually engage in health self-tracking for personal reasons and with individual goals. On the other hand, we need to determine whether there are nonmonetary benefits for that target group that can possibly influence the willingness to donate data positively.

### Research Question and Objectives

We argue that digital forced-choice nudging in general is an appropriate tool to introduce behavioral change toward donating personal health-related data among health self-trackers because health self-trackers are very likely to share their self-tracked data with third parties and primarily have other intentions for tracking. Thus, data donation for research might be perceived as rather unimportant and therefore present an appropriate field for effective nudging.

This quantitative study aims to investigate whether a digital forced-choice nudge can influence the willingness of German health self-trackers to donate self-tracked health-related data for research. We want to contribute to the body of knowledge on the effectiveness of nonmonetary incentives derived from the known needs of the target population. For this, we also consider research on gender differences in privacy tendencies on the web and offline. This shows that women display higher privacy concerns than men. This is in line with both evolutionary and social role theories. Therefore, we analyzed male and female health self-trackers separately [17,18]. Our study thus enables a gender-specific statement on influencing factors on the voluntary donation of personal health data and, at the same time, on the effectiveness of digital nudges for health self-trackers in Germany.

### Background and Theory

#### Nudging

When discussing the active decision to donate self-collected health data and possible contributing factors, we already know that various psychological effects influence individuals, consciously or unconsciously, during their decision-making process [19,20]. People often act impulsively, emotionally, or simply out of habit [21]; they are not always able to calculate the expected consequences of given options and therefore choose the seemingly best available one [22].

In this context, nudges are tools for influencing behavior in decision-making processes without resorting to prohibition, commandments, or economic incentives [19,23,24]. Thus, a nudge is a nonregulatory approach that attempts to motivate individual behavior change through subtle alterations in the choice environments that people face [19,25], whereas a suggested benefit is embedded in the decision-making process [26]. Thus, Karlsen and Andersen [27] define nudging as a term for influencing decisions and behavior using suggestions, positive reinforcement, and other noncoercive means. Löfgren and Nordblom [28] show that the likelihood of a nudge having an effect is higher for choices that the individual perceives as rather unimportant and at a moment in time with limited attention. In summary, the behavior of our study group, health self-trackers in Germany, can potentially be influenced by a nudge that promises a benefit based on existing target group needs and is implemented at a point of limited attention.

#### Nudges in Health Systems

Nudges are widely applied in health systems, with the ultimate goal of a healthy population. According to Holland et al [29], most nudging measures aim at a healthier diet, more exercise, and the reduction of alcohol and tobacco consumption and are thus frequently applied in preventive interventions. Especially in the nutrition field, we find a body of evidence for the effectiveness of nudging interventions: the use of nudges such as food traffic lights, the prominent placement of healthy food alternatives, or the transparent display of calories increases the choice of the healthier option by an average of 16.3% in test participants [30]. Okeke et al [31] investigated the impact of haptic (digital detox) nudging (phone vibration) to reduce the time spent on the web to improve users’ well-being, successfully reducing daily screen time by over 20%. The growing body of evidence on nudging is also increasingly attracting the attention of health care insurance providers. They can potentially realize considerable savings by encouraging their insureds toward targeted (healthier) behavior changes while offering the prospect of bonuses in return.

#### Digital Nudges

With the extensive use of digital devices, decision-making in digital choice environments (so-called digital nudging) has emerged, especially in the field of e-commerce [27,32]. Digital nudges affect value cocreation by (1) widening resource accessibility, (2) extending engagement, or (3) augmenting human actors’ agency [33]. Similar to offline nudges, ethical considerations for digital nudges are already discussed in the literature, focusing on topics such as preserving individuals’ freedom of choice or autonomy, transparent disclosure of nudges, and individual (proself) and societal (prosocial) goal-oriented justification of nudging [34]. Mirsch et al [32] pointed out that digital nudging in general is a promising research area with great potential for improvement and opportunities, especially regarding user interface, user experience, and digital service design questions. The future potential is supported by findings from the study by Hummel and Maedche [35], who discovered that today, only 62% of digital nudging treatments are statistically significant. Regarding the effect size of digital nudges, a quantitative review showed no difference (median effect size of 21% depending on category and context) compared with offline settings while offering new perspectives of individualization [35]. However, research on smart digital nudges in the health care or data donation field is limited. Meske et al [36], for example, conclude that digital nudging in hospitals can positively influence the use of technology, new value creation, changes in structures, and consequently financial dimensions of digital transformation, supporting caregivers as well as caretakers [36]. Regarding charity program participation, forced-choice nudges are found to be the most efficient ones [37,38]; a review reveals that overall default nudges are most effective, and precommitment strategies are least effective [35].

### Hypotheses Development

#### Overview

To test the effectiveness of digital forced-choice nudges for data donation among health self-trackers in Germany, we need to identify the needs of our test group to use the right triggers for behavior change—in our experiment, donate tracked data. The prospect of need satisfaction could be a potentially attractive reward, which might encourage self-trackers to donate their data in return. To this end, we will derive potentially attractive counter values (benefits) for framing in a digital forced-choice nudge based on the known prevailing motives and needs of our target group and for data donation in general.

#### Need of Achievement and Power by Self-expertization

People with a diagnosed disease predominantly track vital signs or biological parameters [39-42]. Intrinsic motivation to improve disease management by advancing personal disease knowledge and controlling health indicators (such as glucose or blood pressure levels) is key for tracking [39-42]. Goals are controlling symptoms and preventing or delaying disease progression.

People with self-perceived disease risk factors mainly track their dietary and physical activities. Motives include potential and subjectively perceived risk prevention (such as obesity) or the desire to learn and promote a healthier lifestyle [43,44]. People without a diagnosed or self-perceived disease or prevalence primarily track their nutrition and exercise parameters. Self-design by performance optimization and monitoring performance progress improvement is the motivation behind [45-47]. A less relevant motive is self-entertainment, which includes natural curiosity, a basic interest, and fun in data collection and visualization [46,48,49].

Health self-trackers thus collectively possess a desire to use digital technologies to optimize health and well-being via self-monitoring [50,51]. Self-motivation, self-discipline, or the desire for performance enhancement are motives found in every user group [52,53] and can be labeled as self-expertization [54]. According to McClelland, these motives arise from the need for achievement and power (over a disease or one’s own body) [55-59]. As self-expertise is key in every subpopulation with no regard to personal medical conditions, our first hypothesis is as follows:

- H1A: The prospect of receiving individualized tips to improve one’s health has a positive influence on female health self-trackers’ willingness to donate personal self-collected health-related data for research.
- H1B: The prospect of receiving individualized tips to improve one’s health has a positive influence on male health self-trackers’ willingness to donate personal self-collected health-related data for research.

#### Need of Self-actualization by Contributing to Society

Research on the motives to donate personal data, for example, to charity, is consistent with findings on motives supporting prosocial behavior, such as blood donation [60-63]. Donations can positively impact self-image and sense of self, as the donor receives appreciation and care in return [64]. Thus, individual needs as well as the need for self-actualization are also satisfied in this context [65]. Kalkman et al [14] pointed out that although participants recognized the actual or potential benefits of data donation for research, they expressed concerns about confidentiality and data abuse [14]. Nonetheless, 2 positive influences on the willingness to donate personal data exist: social responsibility or sense of duty is the first and most influential factor [66]. It refers to indirect reciprocity: giving back to the community and expecting the same treatment in return [67,68]. This altruistic motive is predominantly based on perceived empathy—the willingness to help out of compassion [69].

Second, an individual’s perception of the significance of their own contributions to the community is crucial. Nudging intervention can realize this by emphasizing benefits, such as potentially accelerating the cure of disease by valid research findings and improved therapeutic interventions [16,70].

Derived from identified positive influences on data donation in general and underlying needs, our second hypothesis is as follows:

- H2A: The prospect of contributing to the community has a positive influence on female health self-trackers’ willingness to donate personal self-collected health-related data for research.
- H2B: The prospect of contributing to the community has a positive influence on male health self-trackers’ willingness to donate personal self-collected health-related data for research.

#### Need of Recognition and Social Belonging

According to Gimpel et al [46], motives for sharing self-tracked data, such as diet- and exercise-related or vital and biological parameters, can be referred to as self-association. Cost-benefit trade-offs regarding sharing tracked data are strongly linked to situationally perceived experience [71]. On the basis of the social exchange theory by Homan, users unconsciously or consciously weigh the costs or disadvantages of disclosure against personally perceived advantages [72]. Prospects of satisfying feelings of belonging (to a community) and identification with personalized and individual data outweigh possible disadvantages, for example, receiving personalized advertisements or privacy concerns [46]. Self-association can be traced back to social and individual needs satisfaction according to Maslow and Kruntorad [65], as individuals feel an urge for recognition and belonging and, based on this, possess a desire for esteem and prestige. We derive hypotheses 3 and 4 according to the identified motives for personal health-related data sharing as follows:

- H3A: The prospect of recognition by the peer group (classification of personal tracking results) has a positive influence on female health self-trackers’ willingness to donate personal self-collected health-related data for research.
- H3B: The prospect of recognition by the peer group (classification of personal tracking results) has a positive influence on male health self-trackers’ willingness to donate personal self-collected health-related data for research.
- H4A: The prospect of belonging to a peer group (via personal donation activity) has a positive influence on female health self-trackers’ willingness to donate personal self-collected health-related data for research.
- H4B: The prospect of belonging to a peer group (via personal donation activity) has a positive influence on male health self-trackers’ willingness to donate personal self-collected health-related data for research.

## Methods

### Study and Questionnaire Design

To test hypotheses 1 to 4 using digital forced-choice nudges, we set up a web-based experiment with the questionnaire tool LimeSurvey for health self-trackers in Germany. We generated 5 different mock-ups of the tracking app, Runtastic. We chose the app for its continued popularity, familiarity across all age groups, and duration in the market (since 2009—one of the first apps for health self-tracking) [73-75]. These mock-ups depicted the following situation:

the user has just tracked a run of 5.2 km with Runtastic. This screen in particular is a characteristic of a frequently shared status update on Facebook or Twitter [76]. It represents the sharing of personal physical activity by predominantly recreational athletes via social networks [76]. At this point in the user journey within the app, users only want to see their personal stats (and share them).

On completion, a pop-up window with call to action appeared. The pop-up is a new built-in hurdle before reaching the desired results. The person is in a state of physical exertion, having just completed an intense sports session. Attention and interest in pop-up content could be considered lower at this point [28].

Each pop-up window had a recommendation to the user—donating the tracked data to research, followed by information that motivates and helps him choose the suggested behavior—1 of 4 different nudges (N1 to N4) [27]. Presented nudges refer to hypotheses H1A to H4B. N1 is a framing nudge with egoistic benefits, promising individual tips for data donation based on the need for achievement and power through self-experimentation. N2 promises a contribution to society and is thus our prosocial (framing) nudge based on the need for self-actualization by contributing to society. N3 promises the comparison of one’s own results with other users (social norm framing), as the second egoistic nudge, built on the identified need for recognition. N4 promises joining the data-for-science community, indicated by a badge within the app, and is thus a pseudoprosocial nudge that also uses social norm framing (belonging to a community). The counter value is based on the identified needs of social belonging and the desire for prestige. Finally, N0 is the control group, with no offered counter value.

Willingness to donate was queried by assessing the likelihood of donating on an 11-point scale, ranging from 0% to 100% (How likely are you to click *Donate Now*?). Only one of the pop-up windows was randomly included in each questionnaire. In addition to three sociodemographic parameters, *gender, age*, and *education*, we added 2 items as inclusion criteria to the questionnaire. We started by querying *devices used for tracking health-related data* (*smartphone, smartwatch, fitness tracker*, or *none*), allowing multiple answers as well as the *frequency of accessing the tracked data* (*daily, weekly, monthly, fever*, or *never).*

### Recruitment

The recruitment strategy included digital social media channels, such as Facebook, Instagram, LinkedIn, Xing, and Twitter. Facebook groups dedicated to fitness and nutrition topics as well as Instagram stories of female fitness microinfluencers represented key channels. To question active health self-trackers in Germany that engage with their tracked data, the defined exclusion criteria were (1) if participants never used a tracking device and (2) if participants never actively or consciously tracked any health-related data.

### Data Processing and Statistical Analysis

After collection, the data preparation included cleaning and organizing the raw data set in Excel (Microsoft Inc). We diligently checked for errors to eliminate incomplete questionnaires. Data processing involved encoding text format data into numeric indicator variables. Ultimately, our sample included exclusively ordinal-scaled variables suitable for statistical analysis in SPSS. We divided the sample into female and male participants to examine gender-specific differences.

To perform appropriate statistical analysis for the experimental evaluation, we checked the data distribution. In addition, we checked for variance homogeneity within the 5 different test groups for each gender. To validate experimental hypotheses H1 to H4, we compared our 5 groups with each other for men and women separately. Regarding the data set characteristics, we applied the Kruskal-Wallis and Mann-Whitney *U* tests as nonparametric tests to compare independent samples with homogeneous variances. The Kruskal-Wallis test can evaluate whether there is an actual effect of group affiliation in the first step. With a subsequent post hoc test, we checked which of the groups differed significantly. We used the Dunn-Bonferroni test.

## Results

### Sample

We collected 1091 questionnaires in January and February 2021. Following our defined exclusion criteria, we excluded 5.04% (55/1091) of observations because of participants not using a tracking device and 0.73% (8/1091) of observations because they did not track any health-related data actively or consciously. Furthermore, we removed 9.99% (109/1091) of incomplete questionnaires. The final sample consisted of 919 participants.

Our data set was not normally distributed but left-sided (skewness=0.087) and compressed (kurtosis=−1.431; Table 1).

The sample included 68% (625/919) women and 32% (294/919) men. Overall, 45.1% (414/919) of the participants were aged between 18 and 34 years, 46% (423/919) of the participants were aged between 35 and 54 years, and 8.9% (82/919) of the participants were aged >55 years. Overall, only 0.2% (2/919) of participants did not finish high school. Overall, 4.5% (41/919) of the participants were still in school or high school graduates with no additional formal education. In addition, 43.5% (400/919) of the participants were attending or had graduated from college, and 51.8% (476/919) of the participants were going to or had graduated from a university. In terms of tracking frequency, 85.4% (785/919) of the participants reported daily tracking, 10.8% (99/919) of the participants tracked weekly, 1.4% (13/919) of the participants tracked monthly, and 2.4% (22/919) of the participants tracked less than once per month.

We found that 60.3% (554/919) of our sample used a smartwatch for health self-tracking. Overall, 43.2% (397/919) of the participants used a smartphone, and 33% (303/919) of the participants used a fitness tracker (multiple answers were possible).

The sample (N=919) was divided into 5 test groups with N0 as 186 (20.2%; control group without nudge) and 4 different nudge groups (N1=183, 19.9%; N2=163, 17.7%; N3=199, 21.7%; and N4=188, 20.5%; women and men combined).

Taking gender differences in privacy concerns into account, we split the sample into female (Table 2) and male (Table 3) participants for further analysis.

**Table 1:** Data distribution.

Probability to donate (men + women)		Values
**Participants, n (%)**		
	Valid	919 (100)
	Missing	0 (0)
Value, skewness (SE)		0.087 (0.081)
Value, kurtosis (SE)		−1.431 (0.161)

**Table 2:** Description of the female sample—divided into the 5 test groups (n=625).^a^

Nudge	Participants, n (%)	Value, mean (SD; SE; range; 95% CI)
0	127 (20.3)	41.18 (36.113; 3.205; 0-100; 34.84-47.52)
1	115 (18.4)	43.91 (31.336; 2.922; 0-100; 38.12-49.70)
2	118 (18.9)	47.97 (34.901; 3.213; 0-100; 41.60-54.33)
3	149 (23.8)	45.30 (34.574; 2.832; 0-100; 39.70-50.90)
4	116 (18.6)	34.83 (36.295; 3.370; 0-100; 28.15-41.50)

^a^Total: mean 42.77, SD 34.879; SE 1.395; range 0-100; 95% CI 40.03-45.51.

**Table 3:** Description of the male sample—divided into the 5 test groups (n=294).^a^

Nudge	Participants, n (%)	Value, mean (SD; SE; range; 95% CI)
0	59 (20.1)	47.80 (36.863; 4.799; 0-100; 38.19-57.40)
1	68 (23.1)	48.24 (36.608; 4.439; 0-100; 39.37-57.10)
2	45 (15.3)	63.33 (33.439; 4.985; 0-100; 53.29-73.38)
3	50 (17)	56.20 (34.279; 4.848; 0-100; 46.46-65.94)
4	72 (26.3)	41.25 (35.759; 4.214; 0-100; 32.85-49.65)

^a^Total: mean 50.10, SD 36.112; SE 2.106; range 0-100; 95% CI 45.96-54.25.

### Outcomes of Web-Based Experiment: Nudging Female Health Self-trackers

The female sample contained 625 questionnaires. Regardless of the nudge applied, 77% (481/625) of women surveyed were willing to donate their tracked data for research (with a probability between 10% and 100%).

The Levene test indicates homogeneous variances for female health self-trackers (Table 4).

Using the Kruskal-Wallis test, we examined whether the probability of data donation is the same across the 5 sample groups. As a result, we had to reject the nil hypothesis because significant differences (*P*=.03) exist across at least 2 groups.

**Figure 1:**
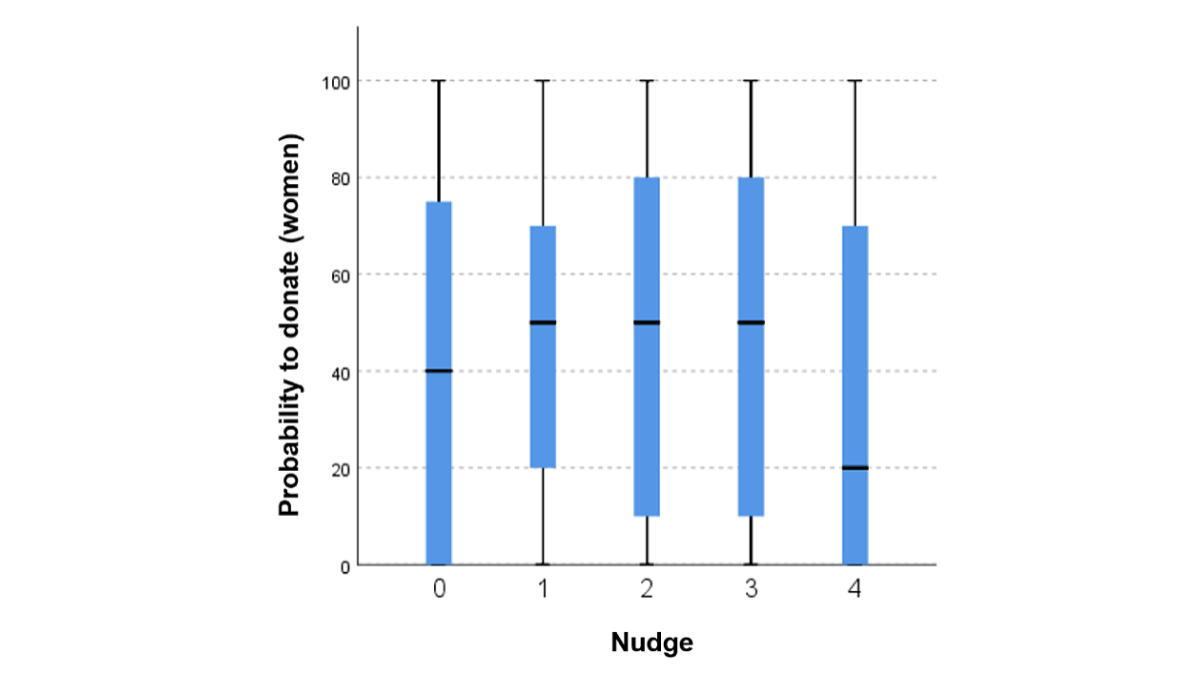
Boxplots for willingness to donate among the 5 test groups (women).

As a post hoc test, we applied the Dunn–Bonferroni test for pairwise group comparison to identify the groups with significant differences. Ultimately, we found no significant difference in the likelihood of donating data between the control group 0 and the 4 test groups. Accordingly, we had to reject the hypotheses H1A, H2A, H3A, and H4A for female health self-trackers, as none of the nudges exerted a significant positive influence on the willingness to donate data. However, there was a significant difference between group 2, the social nudge, and group 4, the prosocial nudge (*P*=.03; Figure 1; Table 5).

**Table 4:** Levene test of homogeneity of variances (women; N=625).

Parameters		Levene statistic (df)	Significance (*P* value)
**Probability to donate (women)**			
	On the basis of the mean	2.007 (4,620)	.09
	On the basis of the median	1.323 (4,620)	.26
	On the basis of the median and with adjusted df	1.323 (4,548.352)	.26
	On the basis of the trimmed mean	1.864 (4,620)	.12

**Table 5:** Pairwise comparison of nudges (women; N=625).

Sample 1-sample 2^a^	Participants, n (%)	Test statistic (SE)	Standard test statistic	Significance (*P* value)	Adjusted significance^b^ (*P* value)
4-0	243 (38.9)	33.037 (22.986)	1.437	.15	.99
4-1	231 (36.9)	52.646 (23.552)	2.235	.03	.25
4-3	265 (42.4)	57.423 (22.161)	2.591	.01	.09
4-2	234 (37.4)	69.431 (23.401)	2.967	.003	.03
0-1	242 (38.7)	−19.609 (23.038)	−.851	.40	.99
0-3	276 (44.2)	−24.386 (21.615)	−1.128	.26	.99
0-2	245 (39.2)	−36.394 (22.884)	−1.590	.11	.99
1-3	264 (42.2)	−4.777 (22.215)	−.215	.83	.99
1-2	233 (37.3)	−16.785 (23.452)	−.716	.47	.99
3-2	267 (42.7)	12.008 (22.055)	.544	.59	.99

^a^Each row tests the null hypothesis that the sample 1 and sample 2 distributions are the same. Asymptotic significances (2-sided tests) are displayed. The significance level is *P*=.05.

^b^Significance values have been adjusted by the Bonferroni correction for multiple tests.

The post hoc test results and plot visualization suggest a potential significant difference between groups 1 and 4 and between groups 3 and 2. We further applied a median-based pairwise group comparison using the Mann–Whitney *U* test. The results indicate significant differences between groups 1 and 4 (N=231; *P*=.01) and between groups 3 and 4 (N=265; *P*=.01).

In summary, for female health self-trackers, no offered counter value, based on egoistic, social, or prosocial motives or needs, has a significant positive or negative influence on the willingness to donate tracked data for research. However, the prospect of showing one’s donation behavior to other users has a negative effect on willingness to donate data in direct comparison with groups that receive a prosocial or a self-serving return for their data donation.

### Outcomes of Web-Based Experiment: Nudging Male Health Self-trackers

Overall, 79.9% (235/294) of the male respondents would donate their data (with a probability between 10% and 100%), regardless of the nudge queried (3% more than female respondents).

The Levene test indicates homogeneous variances for male health self-trackers (Table 6).

Testing for differences among groups in terms of donation probability also revealed a significant difference among male participants (*P*=.02) using the Kruskal-Wallis test.

**Figure 2:**
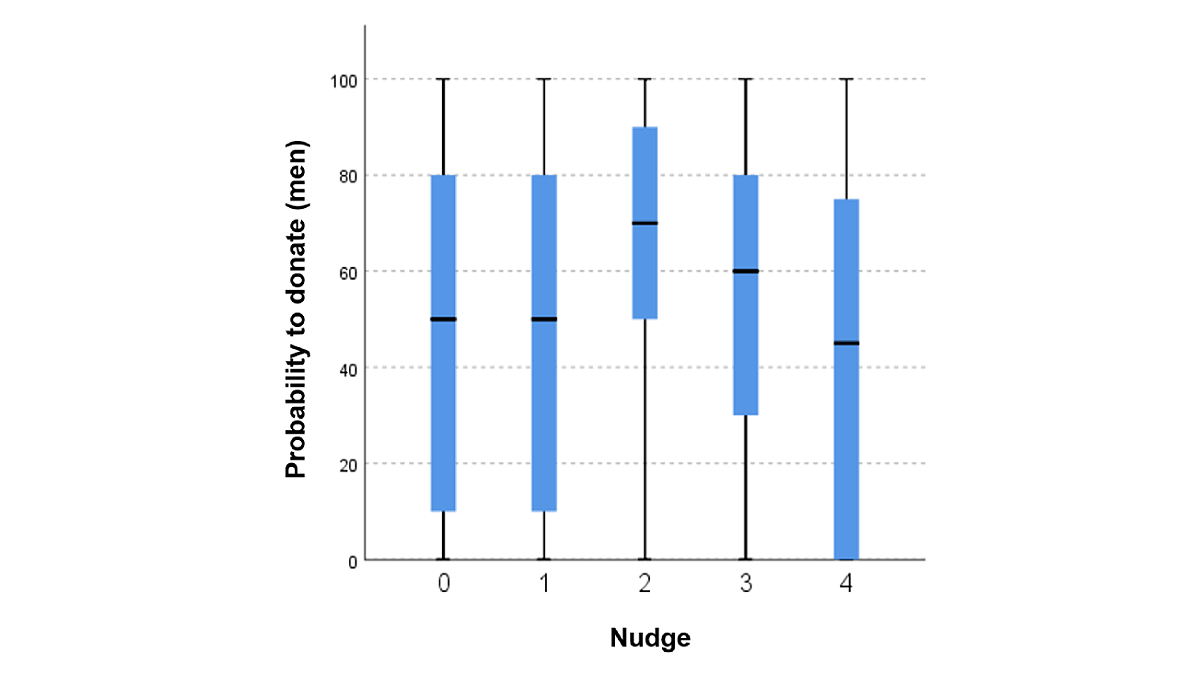
Boxplots for willingness to donate among the 5 test groups (men).

A pairwise group comparison also revealed a significant difference between groups 2 (social nudge) and 4 (prosocial nudge; *P*=.02; Figure 2; Table 7).

The Mann-Whitney *U* test revealed significant differences between groups 1 and 2 (*P*=.04), between groups 0 and 2 (*P*=.04), and between groups 3 and 4 (*P*=.02).

As with female health self-trackers, we had to reject hypotheses H1B, H3B, and H4B for men as well. Thus, the prospect of a self-serving benefit or displaying data donation activity, named pseudoprosocial in our experiment, has no significant positive or negative influence on willingness to donate.

On the other hand, the prospect of making a prosocial contribution significantly influences the likelihood of donating self-tracked (health) data with a small effect size (*r*=0.21; *Z*=−2.087; N=104). Accordingly, we could not reject hypothesis H2B. Comparing means, nudging male health self-trackers in their donating decision-making process with a prosocial nudge would increase the willingness to donate data by 15.5%. At the same time, for male health self-trackers, we found evidence that open data donation significantly reduces the probability of donation compared with a *secret donation* (nudge 2), as well as compared with test groups 1 and 3, receiving a personal benefit. In addition, a group comparison of participants receiving personal tips and participants merely making a prosocial contribution reveals again that prosocial reasons are superior to selfish ones and increase the likelihood of donating data in direct comparisons.

By dividing the sample into male and female participants, we considered gender differences because of the known privacy concern differences between men and women. We also split the sample into different groups regarding age, education, devices used for tracking health-related data, and tracking frequency during analysis. The results show that there are no significant increases or decreases in the willingness to donate self-tracked health-related data when the sample is divided into the stated groups. A correlation analysis did not show any significant positive or negative correlations with the willingness to donate any other variable but gender (*P*=.001).

**Table 6:** Levene test of homogeneity of variances (men; N=294).

Parameters		Levene statistic (df)	Significance (*P* value)
**Probability to donate (men)**			
	On the basis of the mean	0.873 (4,289)	.48
	On the basis of the median	1.127 (4,289)	.34
	On the basis of the median and with adjusted df	1.127 (4,275.393)	.34
	On the basis of the trimmed mean	0.975 (4,289)	.42

**Table 7:** Pairwise comparison of nudges (men; N=294).

Sample 1-sample 2^a^	Participants, n (%)	Test statistic (SE)	Standard test statistic	Significance (*P* value)	Adjusted significance^b^ (*P* value)
4-0	131 (44.6)	15.441 (14.806)	1.043	.30	.99
4-1	140 (47.6)	17.372 (14.258)	1.218	.22	.99
4-3	122 (41.5)	35.604 (15.522)	2.294	.02	.22
4-2	117 (39.8)	50.646 (16.022)	3.161	.002	.02
0-1	127 (43.2)	−1.932 (15.001)	−.129	.90	.99
0-3	109 (37.1)	−20.163 (16.207)	−1.244	.21	.99
0-2	104 (35.4)	−35.205 (16.687)	−2.110	.04	.35
1-3	118 (40.1)	−18.231 (15.708)	−1.161	.25	.99
1-2	113 (38.4)	−33.274 (16.203)	−2.054	.04	.40
3-2	95 (32.3)	15.042 (17.325)	.868	.39	.99

^a^Each row tests the null hypothesis that the sample 1 and sample 2 distributions are the same. Asymptotic significances (2-sided tests) are displayed. The significance level is *P*=.05.

^b^Significance values have been adjusted by the Bonferroni correction for multiple tests.

## Discussion

### Principal Findings and Comparison With Previous Work

As apps represent digital environments in which users have to make decisions on an ongoing basis, a neutral choice presentation is impossible from a behavioral economics point of view. User interface design is crucial and influences users’ app interactions. Developers need to know and understand user decisions and motives, as well as the inferable effects of design on user decision-making, to support desired actions. Our results show that digital nudges addressing the right user needs seem to be an action-triggering operation. Specifically, a digital forced-choice prosocial framing nudge, presented in a pop-up window, can increase the willingness to donate data among male health self-trackers by 15.5%. The results are in line with research on the effectiveness of digital nudging in general (20%) as well as on the effectiveness of offline nudges in health care (16%) [30,35]. We need to further examine how the nudge itself (framing), the presentation (pop-up window vs no pop-up window), and timing of the digital nudge impact nudge effectiveness. In general, however, developers and researchers should consider digital nudges addressing the need for social responsibility when asking for data donation within this specific user group.

The experimental results confirm the findings of previous research on the motives to donate personal data for the health area. Specifically, for health self-trackers, our study confirms the results of Skatova and Goulding [66] and Mujcic and Leibbrandt [68], who found social responsibility or duty to be the strongest predictor of willingness to donate personal data. Our experiment demonstrated that the prospect of doing something good for society positively influences willingness to donate personal health data. Arguably, the act of donating has a positive impact on self-image and sense of self. Unlike blood donation, which provides immediate reciprocal value to the donor in the form of appreciation and caring (during donation; Schiefer [64]) by those present, our results suggest the opposite for health data donation. The public donation of data and its visibility to third parties, especially peers, can potentially discourage health self-trackers from making this donation—they prefer to do so confidentially. Belonging to an ever more similar group within the group of self-trackers, in this case, people who donate their health data have no positive influence on willingness to donate data and more of a negative influence. Showing *charity*, therefore, negatively influences willingness to donate data. This finding should be taken into account when framing a data donation plea, for example, by explicitly referring to donor anonymity.

Compared with polls regarding willingness to donate data, our results show a significantly lower willingness among active health self-trackers. In contrast to population surveys, which put a willingness to donate data at 80% or social media users’ willingness at 95% [15,16], only 41% (377/919) of surveyed health self-trackers would be willing to donate their tracked data to research with a probability above 50% and only 10% (92/919) with 100% probability. This discrepancy suggests that individuals who actively collect health-related data for a particular purpose value it more. It is important to keep this in mind when designing approaches to access health-related, self-tracked data. Research surveying a population cross-section disregards the hypothetical character of questions and answers, and thus, results can be biased and lead to ineffective measures.

Our results also show that women, who are evolutionarily more concerned about protecting their privacy, do not respond to any of the nudges presented adding to the findings by Tifferet [17] and Farinosi and Taipale [18], who found these gender differences, especially in social media users. Regarding the existing gender gap in clinical trials, addressed by Karp and Reavey [77], research needs to investigate which motives, needs, and nudges can increase access to women’s health data equally.

### Limitations

The findings have a number of limitations. Owing to recruiting primarily via social media and fitness influencers, the sample includes a disproportionately large number of younger and higher educated people as well as mostly female compared with male health self-trackers (2/3 to 1/3), which can bias our results.

Thus, the sample may not represent all German health self-trackers. Furthermore, people engaging in social media and following a call to action from influencers on Instagram and from peers in fitness Facebook groups (participating in the experiment in this case) have fewer privacy concerns and are more likely to share their health-related information with others. We did not perform ex ante power calculations to determine the sample size. Future research may therefore repeat the survey with a larger sample via additional recruitment channels to assess the reliability of our findings. In addition, the chosen geographic focus (Germany) might have biased the results because of cultural differences in terms of relevance and general attitudes toward privacy [78]. Further studies could consider international cross-cultural comparisons to verify the validity of our findings for a global app market.

Participants’ nonreproducible or untruthful answers can also limit the results. The reasons could be the chosen and not clearly comprehensible scale levels or phrasing of the individual benefit. Future experiments could also imbed nudges in a different environment (a different app) and use an even more realistic situation with clickable mock-ups. Our hypothetical request for web-based health data donation may not represent reality. Future experiments could implement a real data donation tool within a popular health self-tracking app to verify our results.

### Conclusions

The growing trend toward digital health and increasing acceptance as well as the use of health apps such as fitness trackers, digital check-ups, and nutrition apps will contribute to a significant increase in nudging measures. This study could aid access to health data for research and long-term care improvement.

Although selfish motives do not significantly influence willingness to donate, linking data donation to added societal value could significantly increase the likelihood of donating among male self-trackers by 15.5%. Thus, addressing the need to contribute to society promotes willingness to donate data among male health self-trackers and should be emphasized when designing campaigns to donate health data. The implementation of forced-choice framing nudges within tracking apps presented in a pop-up window can add to the accessibility of user-generated health-related data for research.
